# Simulation and Optimization of the Auxiliary Cathode for Inter-Electrode Discharge Electric Field in Microarc Oxidation

**DOI:** 10.3390/ma16145065

**Published:** 2023-07-18

**Authors:** Pengxiang Lv, Xiaozhou Zhang, Lei Chen, Shixuan Wang, Zhen Wang, Rongguo He, Le Guan

**Affiliations:** 1 School of Mechanical Engineering, Dalian University, Dalian 116000, China; lpx_cn@163.com (P.L.); zxz_2000@163.com (X.Z.); 15140656753@163.com (S.W.); herongguo@dlu.edu.cn (R.H.); haishengtian_dlu@163.com (L.G.); 2 Liaoning Tiansheng Engineering Technology Co., Ltd., Dalian 116001, China; chenleitiandi@163.com

**Keywords:** micro-arc oxidation, aluminum alloy 2A12, edge effect, finite element analysis, auxiliary cathode

## Abstract

Currently, research on the edge effect issue in the micro-arc oxidation process primarily focuses on investigating process conditions and enhancing additives. However, some scholars have utilized finite element analysis software to simulate the edge effect during the simulation process, overlooking the investigation of the morphology of the auxiliary cathode. This study analyzes the growth characteristics of the oxide film on aluminum alloy 2A12 during micro-arc oxidation. Additionally, the inter-electrode discharge electric field is simulated using the finite element analysis method. The auxiliary cathode is optimized to mitigate the influence of the edge effect on the film layer. The findings indicate that employing a cylindrical shape as the auxiliary cathode instead of a rectangular groove leads to an increased thickness of the micro-arc oxidation film. However, it also results in an augmented length of the film layer affected by the edge effect at both ends of the workpiece. Decreasing the distance between the auxiliary cathode and the workpiece surface leads to a higher thickness of the obtained micro-arc oxidation film. Decreasing the length of the auxiliary cathode results in a reduction in both the thickness of the film layer on the workpiece surface and the area affected by the edge effect. Increasing the eccentric cone ratio of the auxiliary cathode enhances the uniformity of the micro-arc oxidation film layer. In this study, we present a novel auxiliary cathode model that incorporates a smaller cylindrical shell at the center and eccentric cone shells on each side. This model has the potential to enhance the optimization rate of the micro-arc oxidation film layer on cylindrical workpieces by 17.77%.

## 1. Introduction

The micro-arc oxidation (MAO) technique has evolved from the traditional anodizing process and is a surface modification technology that enables the in situ growth of ceramic coatings on valve metals and their alloys [1]. The research on micro-arc oxidation can be traced back to the early 1930s when A. Günterschultze and H. Bez proposed the phenomenon of electric breakdown [2] and conducted studies on the spark discharge process on metal surfaces. In 1977, researchers from the former Soviet Union’s Inorganic Chemistry Institute used an alternating voltage mode with a higher operating voltage than anodizing and named this method “micro-arc oxidation” [3]. In the 1970s, Vijh [4] and others proposed the “electron avalanche” model based on the analysis of the mechanism of spark discharge on metal surfaces. Research on micro-arc oxidation in China began in the early 1990s and is still in the research stage [5]. Early reports on micro-arc oxidation in China mainly focused on the structural morphology [6], growth behavior [7], mechanical properties [8], and friction performance [9] of the coatings. In addition to studying the corrosion resistance, thermal protection, and composite coating properties of the micro-arc oxidation layer, there are also relevant reports on its biological [10], catalytic [11], and dielectric insulation properties [12]. Researchers such as Xue Wenbin and Deng Zhiwei have conducted studies on the composition, properties, and growth behavior of micro-arc oxidation ceramic coatings on aluminum [13], magnesium [14], and titanium [15] alloys. Research institutions such as Harbin Institute of Technology and Beijing Normal University have also conducted extensive research on micro-arc oxidation and achieved certain results.

**Figure 1:**
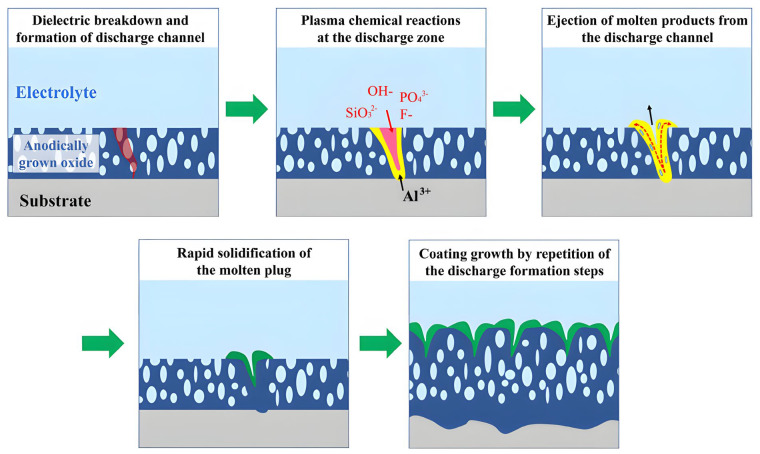
Schematically illustrating the MAO coating formation process [19].

The MAO apparatus comprises essential components, namely a power supply, cooling equipment, and an electrolyte tank. During the process of micro-arc oxidation, the anode of the power supply is typically connected to the metal intended for the treatment, while the cathode is connected to stainless steel, serving as the counter electrode. Upon activation of the power supply, a thin insulating metal oxide film is formed on the surface of the anode metal. As the voltage gradually increases to a critical value, weak points on the metal film are the first to experience a breakdown, resulting in micro-arc discharge and the generation of a highly localized high-temperature region. This region causes the metal substrate and oxide film to melt. Subsequently, when the molten material comes into contact with the electrolyte, rapid cooling occurs, resulting in the formation of a ceramic coating. The breakdown points shift to other weak areas on the metal film, repeating the process until a uniform metal film is formed on the surface of the metal substrate [16]. Research has demonstrated that MAO ceramic coatings exhibit outstanding properties, such as enhanced hardness, wear resistance, and corrosion resistance, in comparison to traditional methods [17,18]. Figure 1 depicts the schematic diagram of the formation process of micro-arc oxidation coating.

Although micro-arc oxidation (MAO) technology exhibits excellent performance, it also encounters significant challenges that necessitate prompt resolution. In the MAO process, localized edge regions, such as the tips and edges of the workpiece, undergo initial discharge, leading to their subsequent convergence towards the geometrically central planes, referred to as the micro-arc oxidation edge effect [20]. The occurrence of the edge effect frequently compromises the quality of the oxide film, resulting in uneven growth, thickness variations, and a decline in film performance. This phenomenon not only leads to failures in MAO processing but also incurs significant losses, posing a crucial problem that demands attention for the industrial application of MAO. Currently, research on this aspect is limited. To mitigate the occurrence of the edge effect and enhance film quality, Min et al. [21] examined the impact of anode-cathode placement patterns on the thickness and uniformity of the oxide film on cylindrical workpieces. They observed that placing the anode horizontally rather than vertically resulted in improved film performance, and the inclusion of an auxiliary cathode enhanced the uniformity of the inner ceramic layer on the cylinder. Xinmeng Zhang et al. [22] investigated the discharge characteristics of confined cathode micro-arc oxidation and found that reducing the inter-electrode distance enhanced the electric field intensity, resulting in higher overall oxidation current and increased film thickness. This improvement in energy efficiency. Furthermore, numerous scholars have investigated the enhancement of film quality by introducing various additives, such as nanoscale ZrO_2_ [23], NH_4_F [24], and Y_2_O_3_ [25], to modify pore size, porosity, composition, and content. However, the micro-arc oxidation edge effect is primarily influenced by electrolyte concentration and electrical parameters. Most studies in this area are still in the experimental phase due to the substantial increase in costs associated with the development of high-power supplies for the technology. Furthermore, achieving universality in optimizing electrolyte and process parameters is challenging because the performance of the oxide film produced by MAO is intricately linked to the specific solution system, concentration, and process parameters employed. The optimization of electrolyte and process parameters can only be accomplished under specific solution systems and process conditions since variations in these conditions will impact the functionality and quality of the resulting film. Conducting a considerable number of experiments not only consumes time but also demands substantial human and material resources.

Given the continuous advancement and maturation of numerical simulation techniques and large-scale simulation software, numerous fields can be explored through numerical simulation. Thus, the integration of numerical simulation techniques into the research process of micro-arc oxidation is essential. Li Huaping et al. [26] estimated the convective heat transfer coefficient on the α-Al_2_O_3_ film surface during the micro-arc oxidation process through experimental measurements and ANSYS simulations. They observed that the Al substrate contributed to heat dissipation during the cooling process, where a considerable amount of heat was dissipated through the adjacent regions of the oxidized alumina or aluminum metal substrate. He Fei [27] employed computer simulation to investigate the film formation process and power supply topology, aiming to analyze the real-time production of micro-arc oxidation films. Tang Yan et al. [28] examined the static electric field in the micro-arc oxidation bath and the distribution of current density on the anode specimen’s surface for various electrode configurations, employing Ansoft Maxwell finite element analysis software. They suggested strategies to mitigate the edge effect, such as (1) employing two parallel plate-shaped anodes and cathodes with infinite length and width, (2) utilizing two concentric cylindrical anodes and cathodes with infinite length, and (3) implementing two concentric spherical anodes and cathodes. Jiang Man [29] conducted numerical simulations of the micro-arc oxidation process on 7075 aluminum alloy, resulting in a film layer with thick edges and a thin center on the surface of the anode specimen. Additionally, the current density exhibited a distribution trend with higher values at the edges and lower values in the center on the workpiece surface. Cao Kening [30] performed a simulation study on edge burning during micro-arc oxidation using ANSYS software. The results revealed that the introduction of an auxiliary cathode enhanced the uniformity of the electric field distribution, leading to a reduction in the size of the burned area.

Controlling the edge effect and ensuring high-quality film layers at the corners of the samples is critical for achieving products that meet the required standards during the preparation process of micro-arc oxidation (MAO) films. Presently, research on edge burning issues during the MAO process predominantly concentrates on investigating process conditions or enhancing additives. Finite element analysis software has been employed by certain scholars to simulate the edge effect, although the condition of the auxiliary cathode remains unexplored. This study aimed to simulate the inter-electrode discharge electric field during the MAO process through the utilization of COMSOL Multiphysics software. We analyzed the feasibility of introducing and optimizing the auxiliary cathode and validated the results through experiments, aiming to provide a solution for the edge effect in the MAO process. Given the intricate nature of film formation, as it involves various processes, including chemical, electrochemical, anodic oxidation, and plasma oxidation, the MAO process is inherently complex [31]. COMSOL Multiphysics, a comprehensive finite element simulation software, finds extensive application in diverse fields, including fluid flow, heat conduction, electromagnetic analysis, and structural mechanics, for engineering research and scientific computations. It allows the simulation of various physical processes, coupling multiple physical fields, and provides a comprehensive and systematic description of physical phenomena [32]. The combination of existing theoretical advancements with experimental validation has substantiated the ability of COMSOL Multiphysics, a multiphysics simulation software, to simulate the MAO process and mitigate the impact of the edge effect on film layers through cathode optimization, thereby influencing the quality of the MAO film.

## 2. Micro-Arc Oxidation Simulation Model

### 2.1. Assumption Model

The finite element analysis conducted in this study is performed under the following simulation conditions: The electrolyte employed is a mixed solution prepared with a 1:3 ratio of NaOH to Na_2_SiO_3_. The anode utilized is a cylindrical specimen composed of 2A12 aluminum alloy, and the auxiliary cathode is a 304 stainless steel electrolytic cell. The micro-arc oxidation process of aluminum alloy in the silicate system involves numerous intricate chemical reactions. The overall reaction equation for micro-arc oxidation can be expressed as follows [33]:

Al(s) + E(aq) → C(s) + G(g) + Q,(1)

In the given equations:

Al: Aluminum alloy

E: Electrolyte

C: Ceramic coating formed during the reaction

G: Gas generated during the reaction

Q: Heat released during the reaction

Based on Kirchhoff’s current law, the currents involved in oxidation and reduction reactions are equal. In the MAO reaction process, we primarily examine two key reactions: the reduction reaction of hydrogen at the cathode and the oxidation reaction of the metal at the anode. These reactions are chosen as the primary focus of the research, while the other reactions are disregarded to ensure computational accuracy.

Cathodic Process:

(2) $${2H}^{+}+2e\to H_{2}↑,$$

Anodic Process:

(3) $${2Al}^{3+}+{3O}^{2-}\to {Al}_{2}O_{3},$$

The electrochemical module of COMSOL Multiphysics provides a variety of physical field interfaces. These interfaces allow for finite element analysis of the transport and reactions of charged ions and neutral particles in the electrolyte, as well as the reactions taking place at the metal surface. Moreover, they can be utilized to describe the current transfer in both the metal and electrolyte. The secondary current distribution interface specifically takes into account electrochemical polarization while disregarding variations in electrolyte composition and concentration polarization. Equation (4) represents the calculation formula for the secondary current distribution.

Secondary Current Distribution Equation:

(4) $$\eta_{m}=\phi_{s}-\phi_{l}-E_{eq,m},$$

In the given equations:
$\eta_{m}$ is the activated overpotentialV$\phi_{s}$ is the electrode potentialV$\phi_{l}$ is the potential of the electrolyte V$E_{eq,m}$ is the equilibrium potential of the reactionV

In the micro-arc oxidation process of 2A12 aluminum alloy, the electrolyte primarily functions to cool and conduct electricity. Due to the presence of an external stirring mechanism, the electrolyte maintains a flowing state, resulting in minimal concentration changes during the reaction. Consequently, variations in electrolyte concentration are not taken into account. The secondary current distribution interface of COMSOL Multiphysics is chosen, and Equation (4) represents the formula for the secondary current distribution within the COMSOL Multiphysics software. Additionally, considering the presence of a cooling system in the micro-arc oxidation process, it can be assumed that the electrolyte temperature remains constant. Hence, the electrolyte temperature is assumed to be consistent at room temperature (T = 20 °C).

### 2.2. Assumption Model

Based on the aforementioned finite element assumptions, the current conduction between the electrode and electrolyte in the micro-arc oxidation process of 2A12 aluminum alloy can be described by Ohm’s law and the current conservation equation.

Ohm’s Law (Equation (5)) and Current Conservation Equation (Equation (6)):

(5) $$\vec{ı}=-F^{2}\sum z_{i}^{2}\mu_{m,i}c_{i}▽\phi_{i}=-\sigma_{l}{▽\phi }_{l},$$

(6) $$▽\cdot i_{l}=Q_{l},$$

In the given equations:
$i_{l}$ is the current density of the electrolyteA/m^2^F is the Faraday constantC/mol$c_{i}$ is the concentration of the electrolytemol/m^3^$\phi_{l}$ is the potential of the electrolyte V$\sigma_{l}$ is the conductivity of the electrolyteS/mμ_m,i_ is the ion mobilitym^2^/(s·J·mol)$z_{i}$ is the charge carried by the ion


### 2.3. Physical Model

**Figure 2:**
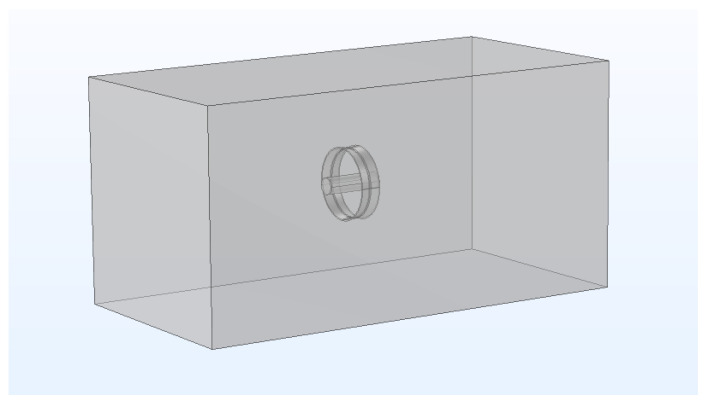
Finite Element Analysis Model of Micro-Arc Oxidation.

A traditional immersion MAO setup typically consists of several key components, including a specialized power supply for MAO, an electrolyte tank (cathode), a workpiece anode, a cooling system, an electrolyte solution, and a stirrer. The stirrer is primarily used to ensure the flow of the electrolyte, maintaining an equilibrium ion concentration and preventing localized depletion of ions caused by the MAO reaction [34]. The cooling system plays a crucial role in dissipating heat and keeping the electrolyte temperature within a reasonable range [35]. The power supply is responsible for providing the required voltage for the micro-arc oxidation process [36]. The functions of these components can be represented by relevant parameters defined in the boundary conditions. Figure 2 illustrates a simplified finite element analysis model of the micro-arc oxidation process, excluding the fixture used for suspending the workpiece.

## 3. Analysis of Simulation Results

Based on the growth mechanism of the micro-arc oxidation coating, it is understood that the coating growth initiates from plasma discharges occurring on the anode surface, which are directly influenced by the electric field on the anode surface. The electric field within the working solution exists between the cathode and the anode. Hence, the size and shape of the auxiliary cathode play a crucial role in determining the quality of the MAO coating growth. Any changes in the size and shape of the auxiliary cathode lead to variations in the current density on the workpiece surface, thereby affecting the thickness of the oxide layer. Equation (7) represents the calculation formula for determining the thickness of the generated oxide layer. In this study, our objective is to optimize the shape and size of the auxiliary cathode based on significant influencing parameters of the coating growth, such as current density, molar mass, aluminum alloy density, current efficiency, and porosity mentioned in the thickness formula. The ultimate goal is to achieve a uniform and dense micro-arc oxidation coating.

Thickness Equation:

(7) $$\frac{\left[cd.itot\;*\;T\left[min\right]\;*\;M\;*\;eff\right]}{\left[\sigma \;*\;F\_const\;*\;rho\;*\;(1-pro)\right]}$$

In the given equations:
cd.itot: current densityM: the molar mass of aluminumT: time parametereff: current efficiencyσ: coefficientF_const: Faraday’s constantrho: the density of the aluminum oxidepro: porosity

### 3.1. Comparison between the Original Discharge Model and the Cylindrical Auxiliary Cathode Model

**Figure 3:**
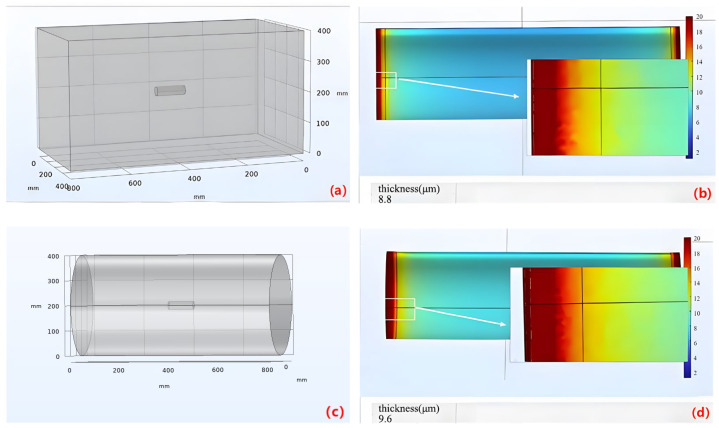
(**a**) Original Discharge Model; (**b**) Simulation Results; (**c**) Cylindrical Discharge Model; (**d**) Simulation Results.

The initial discharge model utilized a conventional rectangular cathode, as illustrated in Figure 3a. Due to the relatively larger size of the cathode compared to the workpiece and the greater distance between the cathode and the workpiece, the inter-electrode electric field between the cathode surfaces and the workpiece is weak and exhibits minimal variation. As a result, even though the cathode is in the form of a rectangular cavity, the distribution of current density on the workpiece surface is mainly influenced by the edge effect. To achieve a uniform electric field distribution on the cylindrical surface of the workpiece, considering the workpiece’s cylindrical shape, a cylindrical auxiliary cathode was introduced near the workpiece, as depicted in Figure 3c. This modification aims to improve the uniformity of micro-arc discharge during the MAO process, especially in the central region of the workpiece, thereby resulting in a more uniform MAO coating on the workpiece surface. The simulation results are illustrated in Figure 3d.

The average thickness of the oxide layer on the workpiece surface in the original discharge model is 8.8, whereas, in the cylindrical auxiliary cathode model, it is 9.6. This implies that the use of the cylindrical auxiliary cathode model leads to a greater thickness of the oxide layer compared to the original discharge model. Hence, replacing the original rectangular groove model with the cylindrical model demonstrates an optimization effect.

### 3.2. The Impact of the Distance between the Auxiliary Cathode and the Workpiece on the Oxide Layer

#### 3.2.1. The Relationship between Average Oxide Layer Thickness and Auxiliary Cathode Radius

**Figure 4:**
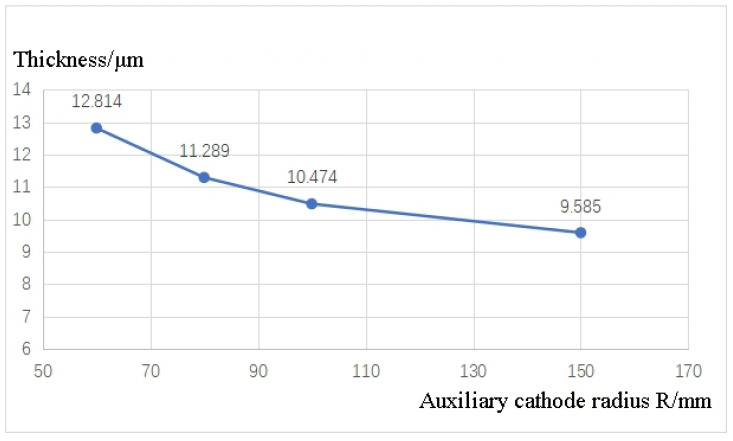
Variation of the average surface oxide layer thickness h1 with R.

Changes in the distance between the electrodes lead to variations in the electric field distribution between the anode and cathode, thereby impacting the distribution of current on the electrode surface. Higher current densities promote rapid growth of the oxide layer, which consequently affects the uniformity of the micro-arc oxidation layer generated on the specimen surface. Thus, the interelectrode distance between the auxiliary cathode and the workpiece surface plays a significant role in determining the current density, which directly influences the thickness of the micro-arc oxidation layer. In this study, the interelectrode distance was adjusted by altering the radius of the auxiliary cathode. Simulations were conducted for different values of R, and the relationship between the average thickness of the oxide layer on the workpiece surface and R is illustrated in Figure 4.

The simulation results indicate that reducing the radius (R) of the auxiliary cathode leads to an increase in the average thickness of the oxide layer (h1) on the workpiece surface. However, it is important to consider the intense plasma discharge, heat release, and bubble overflow phenomena that occur during the micro-arc oxidation process. Experimental observations have shown that the vigorous reaction primarily occurs within a distance of approximately 30 mm from the workpiece surface. Therefore, continuously decreasing the interelectrode distance (R) solely to increase the oxide layer thickness (h1) is not advisable. It is crucial to maintain effective fluid circulation, reduce the temperature in the reaction zone, and protect the auxiliary cathode. At an interelectrode distance of R = 60 mm, the oxide layer thickness (h1) is measured to be 12.8 μm, representing a 38.3% increase compared to the original discharge model’s thickness of 8.83 μm. This considerable enhancement in layer thickness and growth efficiency suggests that R = 60 mm is a favorable choice for further investigation.

#### 3.2.2. Experimental Verification of the Influence of the Distance between the Auxiliary Cathode and the Workpiece on the Oxide Layer

**Figure 5:**
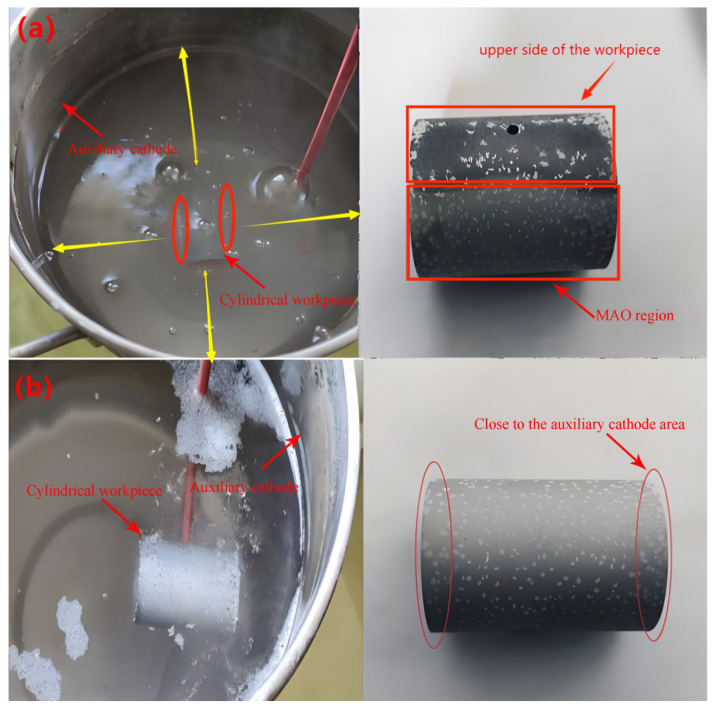
(**a**) Experimental Setup (**left**) and Reaction Results (**right**) for Experiment 1; (**b**) Experimental Setup (**left**) and Reaction Results (**right**) for Experiment 2.

In Section 3.2.1, it was mentioned that reducing the distance between the auxiliary cathode and the workpiece leads to an increase in the thickness of the oxide layer. This suggests that the reactions occurring closer to the auxiliary cathode are more vigorous, as observed in experiments. The purpose of this section is to experimentally verify that reducing the distance between the auxiliary cathode and the workpiece results in more intense reactions at the end closer to the auxiliary cathode. Figure 5a depicts Experiment 1, where the workpiece is positioned in the middle of the auxiliary cathode. This experiment demonstrates that the left-right and front-back distances between the workpiece and the auxiliary cathode are nearly identical, while the lower side of the workpiece is closer to the auxiliary cathode compared to the upper side. Figure 5b corresponds to Experiment 2, where the workpiece is shifted to the right, bringing it closer to the auxiliary cathode. The Figure 5b (left) shows that the right end of the workpiece is in closer proximity to the auxiliary cathode.

The results of Experiment 1 demonstrate that the micro-arc oxidation (MAO) regions on the left and right sides of the workpiece, as well as the lower side, are more pronounced. This is due to higher current density and more discharge energy in these regions, closer to the auxiliary cathode. However, the upper side, which is farther from the auxiliary cathode, exhibits less distinct MAO regions due to lower current density and less discharge energy in the middle of the workpiece. In Experiment 2, as shown in the left image, the right side of the workpiece shows a higher concentration of bubbles and micro-arcs compared to the left side. This indicates more intense MAO reactions on the right side, which is closer to the auxiliary cathode. The right image demonstrates a more uniform oxide layer in the region closer to the auxiliary cathode compared to the left side. By conducting Experiments 1 and 2, it is verified that reducing the distance between the auxiliary cathode and the workpiece leads to more intense MAO reactions. Moreover, the oxide layer in the region closer to the auxiliary cathode appears denser, consistent with the finding in Section 3.2.1 that reducing the distance between the auxiliary cathode and the workpiece increases the average thickness of the oxide layer. Therefore, the simulation results align with the experimental observations, confirming the relationship between the distance to the auxiliary cathode, the intensity of MAO reactions, and oxide layer thickness.

### 3.3. The Relationship between Average Oxide Layer Thickness and Length of the Auxiliary Cathode

**Figure 6:**
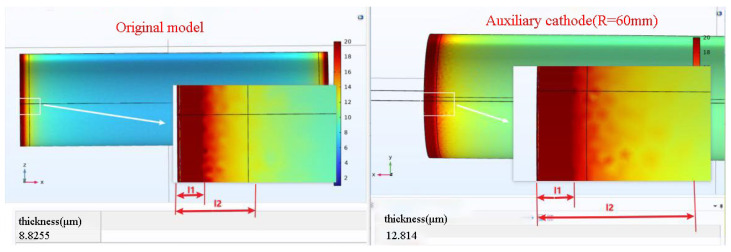
Comparison of the Original Discharge Model (**left**) and Simulation Results for R = 60 mm (**right**).

As mentioned in Section 3.2, reducing the radius (R) of the auxiliary cathode leads to an increase in the average thickness (h1) of the oxide layer on the workpiece surface. However, when R is set to 60 mm, both the tip effect influence region (l1) and the tip effect diffusion region (l2) are larger compared to the corresponding regions in the original discharge model, as shown in Figure 6. Although reducing the electrode spacing effectively increases the average thickness of the oxide layer, it also results in an increased non-uniformity of the local micro-arc oxidation (MAO) layer. This indicates the need for further optimization of the auxiliary cathode design to reduce the size of the tip effect influence region (l1) and the tip effect diffusion region (l2).

**Figure 7:**
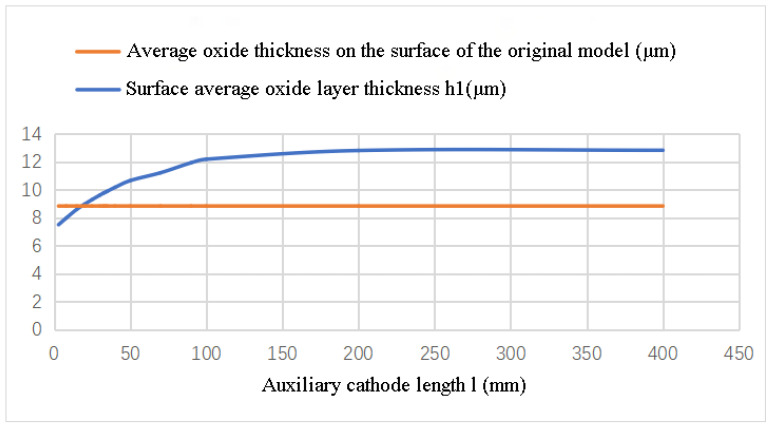
Variation of the Average Oxide Layer Thickness (h1) with the Length of the Auxiliary Cathode (l).

To shield the interference of external stray electric fields, the length of the auxiliary cathode is designed to be significantly greater than that of the workpiece. However, the additional length at both ends of the auxiliary cathode can lead to an increased tip effect on the workpiece surface. To mitigate this effect, it is necessary to appropriately decrease the length (l) of the auxiliary cathode. Simulation results demonstrate that the average thickness (h1) of the oxide layer increases with an increase in the length (l) of the auxiliary cathode, as shown in Figure 7. However, when the length (l) is less than 20 mm, the average thickness (h1) is below the average oxide layer thickness (8.83 μm) of the original discharge model. Therefore, to achieve optimization, the cathode length (l) should be greater than 20 mm.

### 3.4. The Relationship between Optimization Rate and Variation in Length of the Auxiliary Cathode

As analyzed above, the surface current density of the workpiece is primarily influenced by the electrode gap distance (R) and the length (l) of the auxiliary cathode. Therefore, by setting R to 60 mm, the goal of changing the oxide layer thickness can be achieved by adjusting only the length (l) of the auxiliary cathode. In this section, the impact of the auxiliary cathode length (l) on the optimization effect of the oxide layer thickness is analyzed using the optimization rate Equation (8).

Equation *Y* for Optimization Rate of the Film Thickness:

(8) $$Y=\left\{\left[(\frac{a-l1}{a})\;*\;\gamma +(\frac{b-l2}{b})\;*\;\gamma \right]\;*\;\alpha +(\frac{l3-c}{c})\;*\;\beta \right\}\;*\;100\%,$$

In the given equations:

*α*, *β*, *γ*: the proportionality coefficients

a, b, c: the parameters of the original discharge model

l1,l2,l3: the values corresponding to different lengths of the auxiliary cathode

**Figure 8:**
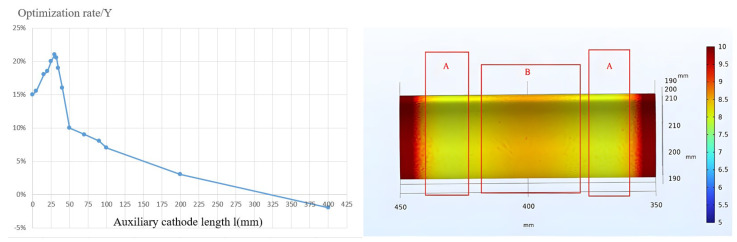
Optimization Rate (Y) of Film Thickness and Simulation Results for Auxiliary Cathode Lengths (l) within the range of 3 to 300 mm, with a specific case of l = 33 mm.

The optimization rate of the oxide layer thickness reaches a maximum value of 21.5% when the length (l) of the auxiliary cathode is approximately 33 mm, as depicted in Figure 8. Therefore, a length (l) of 33 mm demonstrates a significant optimization effect. Nonetheless, the resulting oxide layer on the workpiece surface remains non-uniform despite employing the cylindrical auxiliary cathode model. On the central region of the workpiece’s external surface, zones A and B display distinguishable color variations, with zone B appearing darker, indicating a greater thickness of the surface oxide layer. Both zones A exhibit lower thickness values in comparison to zone B. The non-uniformity in the oxide layer arises from the proximity of zone B to the auxiliary cathode, resulting in a more vigorous micro-arc oxidation process in zone B, leading to higher thickness values. Thus, to attain a uniform distribution of thickness values in both zones A and B and achieve a homogeneous micro-arc oxidation layer on the workpiece surface, it is imperative to optimize the structure of the auxiliary cathode. Simulation and verification results indicate that by strategically increasing the separation distance between zone B and the auxiliary cathode, the thickness values in both zones A and B can be rendered more uniform.

### 3.5. The Relationship between Average Oxide Layer Thickness and Length of the Auxiliary Cathode

**Figure 9:**
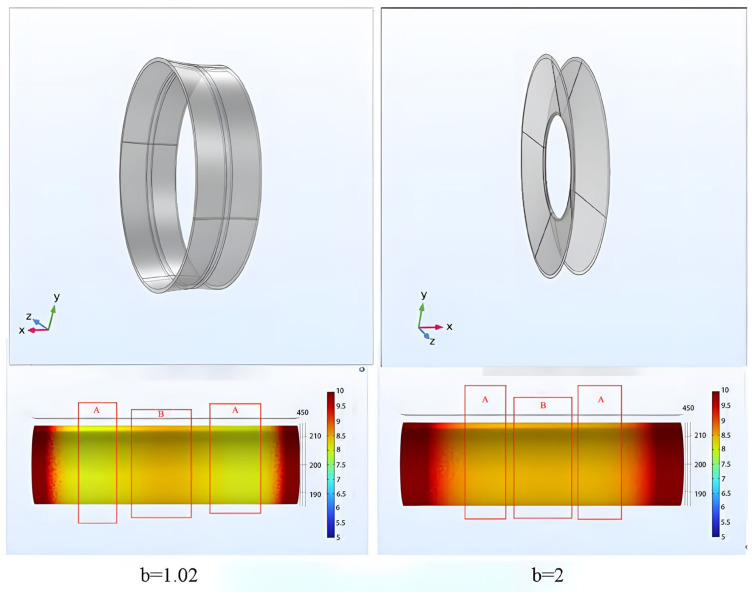
Comparison of Eccentric Cone Ratios b = 1.02 (**left**) and b = 2 (**right**).

The optimized configuration of the auxiliary cathode is depicted in Figure 9, comprising a combination of a cylinder and two eccentric truncated cones. The central portion is a cylinder with a radius (R) of 60 mm and a length (l) of 3 mm. On either side, symmetric eccentric truncated cones are incorporated with the auxiliary cathode length set to 33 mm, which yields the highest optimization rate; the height of the eccentric truncated cones is designated as 15 mm, ensuring a total auxiliary cathode length of 33 mm. By adjusting the distance between the workpiece and the auxiliary cathode, the thickness of the oxide layer on the workpiece surface can be controlled. Therefore, by modifying the ratio (b) of the eccentric cones, different inclinations are achieved, subsequently altering the distances between the auxiliary cathode and zones A and B. As a result, the oxide layer in zones A and B can be adjusted, optimizing the uniformity of these regions. Simulation results demonstrate that employing an eccentric truncated cone ratio (b) of two produces a relatively uniform oxide layer on the central surface, whereas a value of 1.02 for (b) leads to a higher oxide layer thickness in zone B compared to zone A. Thus, modifying the eccentric truncated cone ratio (b) allows for the adjustment of uniformity, as stated earlier. To assess the effectiveness of this optimization, the variance formula (Equation (9)) is utilized to calculate the variances of the average oxide layer thicknesses in zones A and B (h2 and h3) and their combined regions (h4). This enables the evaluation of the impact of the eccentric truncated cone ratio (b) on variance and, consequently, the assessment of its influence on the uniformity of zones A and B.

Equation for Variance *S*:

(9) $$S=\frac{{\left(h2-h4\right)}^{2}+{\left(h3-h4\right)}^{2}}{2}\beta$$

In the given equations:

h2,h3,h4: The Average Oxide Layer Thickness of A, B, and A and B

β: the proportionality coefficient

**Figure 10:**
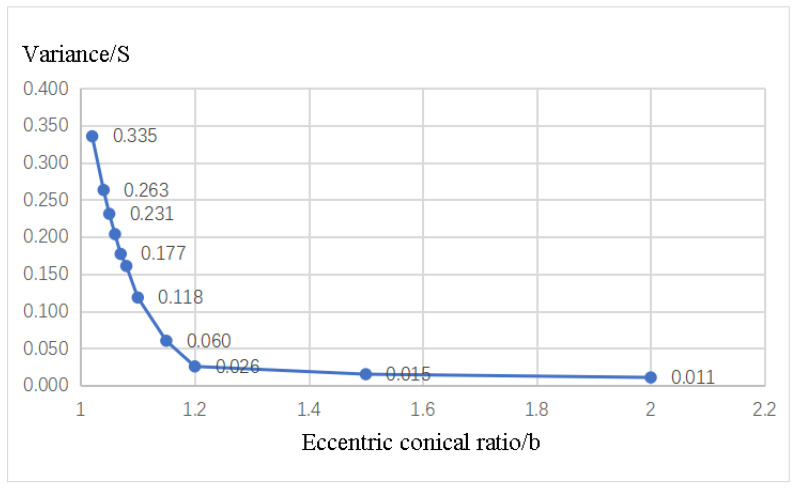
Variation of Variance (S) with Eccentric Cone Ratio (b).

The simulation results demonstrate that as the eccentric truncated cone ratio (b) increases within the range of 1 to 2, the variance (S) value decreases. This finding implies that a higher eccentric truncated cone ratio (b) leads to a more uniform oxide layer in zones A and B, as depicted in Figure 10. Therefore, it can be concluded that increasing the eccentric truncated cone ratio (b) is beneficial for achieving a more uniform oxide layer.

### 3.6. The Relationship between Eccentric Cone Ratio and Optimization Rate of Film Thickness

**Figure 11:**
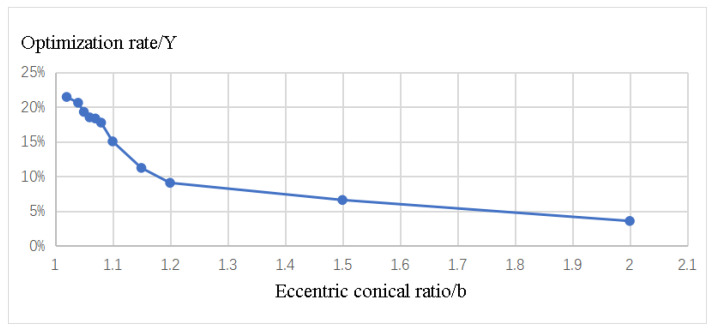
Variation of Optimization Rate (Y) with Eccentric Cone Ratio (b).

As stated in Section 3.2, it is established that increasing the distance between the auxiliary cathode and the workpiece surface results in a reduction in the oxide layer thickness. Therefore, it can be inferred that with an increase in the eccentric truncated cone ratio (b), the overall thickness of the oxide layer on the workpiece surface is expected to decrease. Hence, it is imperative to investigate the behavior of the oxide layer on the workpiece surface under different eccentric truncated cone ratios (b). The simulation plot in Figure 11 illustrates the optimization rate (Y) as a function of the eccentric truncated cone ratio (b).

The graph in Figure 11 illustrates that as the eccentric truncated cone ratio (b) increases, the average thickness of the oxide layer (h1) on the workpiece surface decreases. However, this decrease is accompanied by a reduction in the range of the tip effect influence area (l1) and the tip effect diffusion area (l2).

In conclusion, increasing the eccentric truncated cone ratio (b) improves the uniformity of regions A and B, resulting in a decrease in the variance (S) value. However, this improvement comes at the expense of a decrease in the optimization rate (Y). Therefore, when pursuing uniformity, there may be a compromise in the optimization rate. Consequently, it is crucial to comprehensively consider both the optimization rate (Y) and the variance (S) values to determine the optimal parameter for the auxiliary cathode. To achieve this, the following scoring formula (10) is employed to determine the parameter that strikes a balance between optimization rate and uniformity.

The Scoring Equation *P*:

(10) $$P=(Y\;*\;\delta_{1}-S\;*\;\delta_{2})k,$$

In the given equations:

*Y*: Optimization Rate

*S*: Variance

$\delta_{1},\delta_{2},k$: the proportionality coefficients

**Figure 12:**
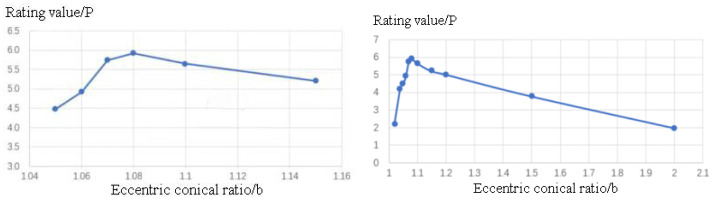
Variation of Scoring Value P with Eccentric Cone Ratio b, and the Range of Eccentric Cone Ratio b from 1.04 to 1.16.

Figure 12 presents the relationship between the scoring values and the eccentric truncated cone ratio (b). It is evident from the graph that the highest scoring value is attained at b = 1.08, within the range of 1.04 to 1.16. This signifies that the auxiliary cathode model with b = 1.08 is the optimal choice, striking a balance between the oxide layer thickness on the workpiece surface and uniformity.

**Figure 13:**
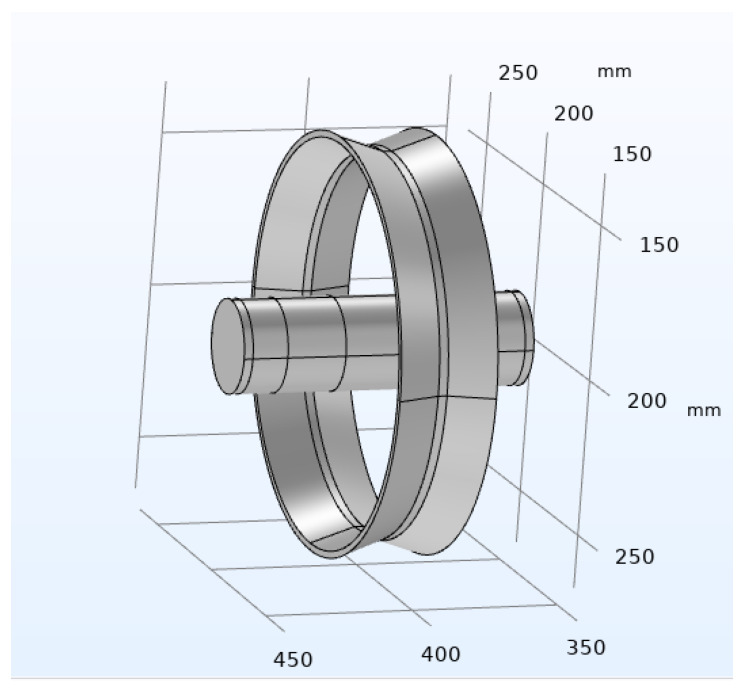
Schematic Diagram of the Optimized Model for the Auxiliary Cathode.

Based on the analysis conducted, it has been determined that the eccentric truncated cone ratio (b) of 1.08 yields the highest scoring value, indicating that b = 1.08 serves as an optimal parameter that strikes a balance between the optimization rate and variance. A comparison of the simulated current density distribution between the original and final models reveals that the current density distribution on the workpiece surface is significantly more uniform in the optimized auxiliary cathode model. The original model’s characteristic of high density at the tip and low density in the middle is no longer observed. This suggests that the micro-arc oxidation reaction on the workpiece surface tends to be more uniformly distributed overall in the optimized auxiliary cathode model, and the influence of the tip effect on the micro-arc oxidation reaction is reduced, resulting in a more uniform oxide layer. The final dimensions of the auxiliary cathode are as follows: a total length of 33 mm, a cylindrical shell with a radius of 60 mm, a length of 3 mm in the middle, and two eccentric truncated cone structures on both sides with a length of 15 mm and a ratio of b = 1.08, as depicted in Figure 13. The optimization rate of the micro-arc oxidation reaction for this auxiliary cathode model, compared to the rectangular groove model, amounts to 17.77%.

## 4. Experimental Validation and Characterization

**Figure 14:**
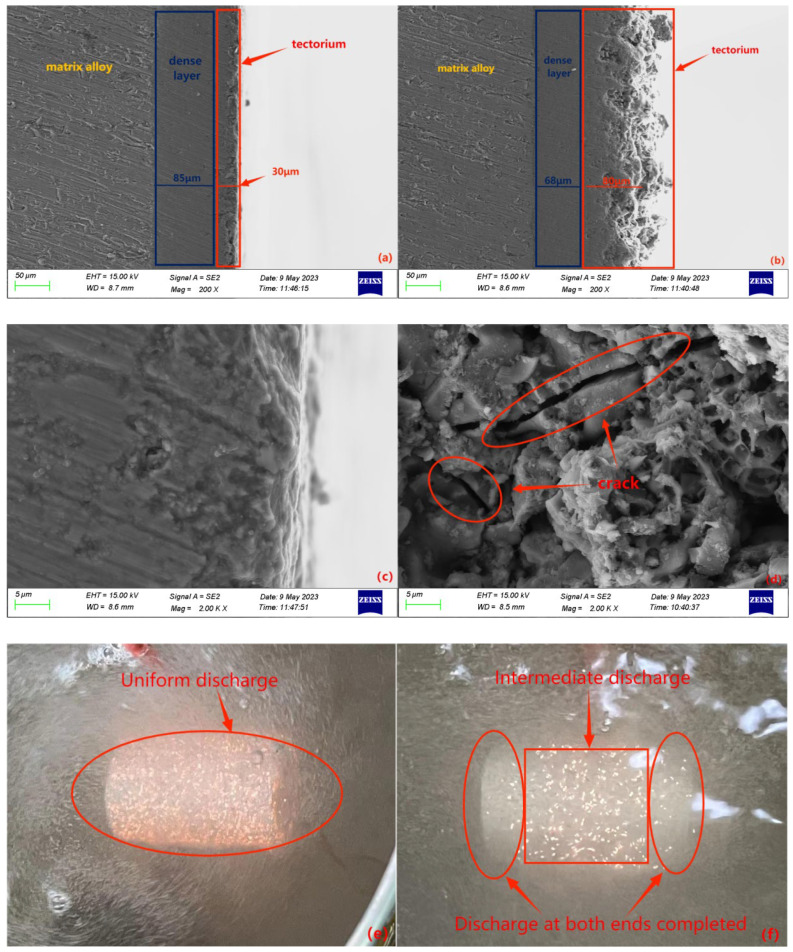
Cross-sectional morphology of the assisted cathode workpiece at both ends (**a**,**c**) and the corresponding reaction process (**e**). Cross-sectional morphology of the unassisted cathode workpiece at both ends (**b**,**d**) and the corresponding reaction process (**f**).

An optimized auxiliary cathode was utilized for the micro-arc oxidation reaction on the exterior of a cylindrical workpiece composed of 2A12 aluminum alloy. The workpiece had a diameter of ∅30 mm and a length of 100 mm. In comparison, another cylindrical workpiece without an auxiliary cathode underwent micro-arc oxidation directly in a rectangular groove. The results demonstrate that the cylindrical workpiece with the auxiliary cathode exhibited uniform discharge without any tip effect at both ends (Figure 14e). The growth of the oxide layer was synchronous, resulting in a uniform thickness and excellent properties. Conversely, the cylindrical workpiece without the auxiliary cathode displayed a tipping effect, with discharges occurring first at both ends and then in the middle (Figure 14f). The growth of the oxide layer was asynchronous, leading to thicker layers at the ends and thinner layers in the middle. Over time, erosion occurred at the ends while the oxide layer in the middle had not fully developed, resulting in a decline in the performance of the oxide layer. Figure 14a illustrates the cross-section of the oxide layer at both ends of the cylindrical workpiece with the auxiliary cathode, revealing uniform growth of the oxide layer, a thicker dense layer, a smaller porous layer, and even growth of the porous layer without erosion (Figure 14c). Figure 14b displays the cross-section of the oxide layer at both ends of the cylindrical workpiece without the auxiliary cathode, demonstrating a decrease in the thickness of the dense layer and an increase in the thickness of the porous layer due to tip discharges. The porous layer experienced accumulation and cracks due to erosion, resulting in poor uniformity of the oxide layer and significantly compromising its performance (Figure 14d).

In conclusion, the utilization of an auxiliary cathode greatly enhances the micro-arc oxidation reaction of cylindrical workpieces, leading to the formation of uniformly dense and high-performance oxide layers.

## 5. Conclusions

1. In this study, we investigate a cylindrical workpiece made of 2A12 aluminum alloy. Through simulation and experimental verification of the micro-arc oxidation process, we propose an effective method to mitigate the tip effect by modifying the morphology of the auxiliary cathode. This research provides a foundation and practical approach for the design and development of the electrode structure in micro-arc oxidation systems.
2. For cylindrical workpieces, we design the auxiliary cathode as a combination of a small cylindrical shell in the middle and eccentric conical shells on each side. This configuration enhances the density and uniformity of the micro-arc oxidation film on the workpiece.
3. During the auxiliary cathode design, we observed that decreasing the distance between the auxiliary cathode and the workpiece surface increases the thickness of the oxide layer. Similarly, reducing the length of the auxiliary cathode decreases the thickness of the oxide layer on the workpiece surface and minimizes the area affected by the tip effect. Moreover, increasing the eccentric cone ratio improves the uniformity of the oxide layer. This provides important ideas for the rational design of the auxiliary cathode.
4. Optimization design based on finite element analysis proves to be an effective approach in modern engineering. Leveraging finite element analysis software enables the anticipation of possible test outcomes in advance, thereby reducing the number of experiments and iterations required to adjust various parameters. This approach significantly enhances efficiency, resulting in cost savings during the design and development process.

## Data Availability

Not applicable.
